# Enhanced survival in resectable duodenal adenocarcinoma with adjuvant chemotherapy: evidence from a retrospective study

**DOI:** 10.3389/fonc.2026.1730374

**Published:** 2026-04-28

**Authors:** Junbao Liu, Yuting Fang, Guifu Wu, Na Liu, Deying Bi, Yibin Xie, Yuxin Zhong, Jianwei Zhang, Dongbing Zhao, Yongkun Sun

**Affiliations:** 1 Department of Medical Oncology, Beijing Chaoyang SanHuan Cancer Hospital, Beijing, China; 2 National Cancer Center/National Clinical Research Center for Cancer/Cancer Hospital, Chinese Academy of Medical Sciences and Peking Union Medical College, Beijing, China

**Keywords:** adjuvant chemotherapy, CA199 level, duodenal adenocarcinoma, overall survival, recurrence-free survival

## Abstract

**Background:**

Duodenal adenocarcinoma (DA) is a rare malignancy, and the effectiveness of adjuvant chemotherapy on survival after surgical intervention remains ambiguous. This study investigates the impact of adjuvant chemotherapy on overall survival (OS) and recurrence-free survival (RFS) in patients with resectable DA.

**Materials and methods:**

Data from ninety-eight stage I-III DA patients who underwent surgical resection between January 1998 and December 2021 at two Chinese institutions were analyzed retrospectively. Survival outcomes were assessed using the Kaplan-Meier method, and multivariable Cox proportional hazards models identified significant prognostic factors.

**Results:**

Of the 98 patients, 45 received adjuvant chemotherapy and 53 did not. The median follow-up was 49 months. Patients who had adjuvant chemotherapy showed a longer median OS (52.9 vs. 25.1 months, p=0.003) and RFS (38.2 vs. 9.9 months, p<0.001) than those without. N stage was associated with reduced OS and RFS. Multivariate analysis identified adjuvant chemotherapy, N stage, and CA199 as independent predictors for RFS and OS. For patients with initial CA199 ≥ 27U/mL, adjuvant chemotherapy appeared to be associated with improvements in RFS and OS, alongside suggestive reductions in recurrence and mortality risks.

**Conclusion:**

Adjuvant chemotherapy correlates with an extended median OS and RFS, as well as a reduced risk of recurrence and mortality in patients who have undergone surgical resection for DA. Specifically, patients with DA who are in the N1 or N2 stage, or those with a CA199≥27U/mL at their initial diagnosis, may show potential clinical benefits from adjuvant chemotherapy as a preliminary exploratory finding.

## 1 Introduction

Small bowel adenocarcinoma (SBA), a rare gastrointestinal malignancy, accounts for less than 5% of all malignant digestive system tumors. In 2022, approximately 11,790 new SBA cases were reported in the United States, resulting in 1,960 deaths (1). Duodenal adenocarcinoma (DA) is the predominant subtype of SBA, comprising over 50% of SBA cases despite representing less than 0.5% of all gastrointestinal malignancies (2).

The main treatment for local (i.e., stages I–III) SBA is surgical resection, including en-bloc lymph node removal. Treatment for DA varies based on tumor location, typically involving pancreaticoduodenectomy for proximal tumors and segmental resection for distal tumors. In cases of unresectable SBA, neoadjuvant therapy incorporating fluoropyrimidine or platinum-based chemotherapy, sometimes in combination with radiotherapy, is recommended (3).

The prognosis is poor, with a 5-year overall survival(OS) rate of 14–33% for all patients and 40–60% for those who are curatively resectable (1, 4). A significant proportion of patients undergoing curative resection experience recurrence (5), underscoring the need for effective adjuvant strategies to enhance OS and reduce recurrence rates. Adjuvant chemotherapy, particularly regimens such as FOLFOX (folinic acid, fluorouracil, and oxaliplatin), CAPEOX (capecitabine and oxaliplatin), 5-FU/LV (5-fluorouracil and leucovorin), or capecitabine alone (3), in SBA remains controversial due to the mixed results from retrospective studies, meta-analyses, and the scarcity of randomized controlled trials (RCTs). While some studies indicate the benefits of adjuvant therapy (chemotherapy or chemoradiotherapy) (6, 7), others show no significant impact or equivocal results (8–11). Notably, most research has not specifically addressed DA, nor has it substantially focused on the response of the Chinese population to these adjuvant therapies.

Given this context, the principal aim of our study is to conduct a focused assessment of the effects of adjuvant chemotherapy on survival outcomes in Chinese patients with resectable DA. Additionally, this study seeks to identify the specific patient cohort that is most likely to benefit from adjuvant chemotherapy, thereby contributing valuable insights to current clinical practices.

## 2 Materials and methods

### 2.1 Data source and patient selection

Patients diagnosed with duodenal adenocarcinoma (DA) who underwent curative resection at the Department of Medical Oncology at Chaoyang Sanhuan Cancer Hospital and the National Clinical Research Center for Cancer were enrolled between January 1998 and October 2021.The main inclusion criteria were as follows: 1)Age > 18 years;2)Pathologically confirmed diagnosis of DA;3)No evidence of distant metastasis;4)Complete R0 resection with regional lymph node dissection;5)No evidence of other concurrent malignancies;6)No adjuvant chemotherapy or completion of at least four cycles of adjuvant chemotherapy;7)Adjuvant chemotherapy initiated within 3–4 weeks after surgery;8)No severe postoperative complications that delayed adjuvant chemotherapy beyond 4 weeks. Patients were excluded if they had incomplete prognostic data, unavailable medical records, R1 resection, or severe postoperative complications precluding timely adjuvant chemotherapy. The study was approved by the ethics committee of Beijing Chaoyang District SanHuan Cancer Hospital(SH-2023020, 2023.7.17), and informed consent was waived due to the retrospective nature of the study.

### 2.2 Data variables

A comprehensive review of patient medical records was conducted to gather demographic data, tumor characteristics, and survival outcomes. The analysis included demographic variables such as age at diagnosis, gender, and the ECOG performance status. In terms of tumor features, TNM stage(version8), the histological grade, the T stage, and the N stage and tumor location in the duodenum (D1: bulb, D2: descending, D3: horizontal, D4: ascending) were recorded. Surgical interventions were analyzed, detailing the number of lymph nodes examined. The presence of perineural, lymphatic, and vascular invasion was also recorded. The use of adjuvant chemotherapy was documented, noting the specific regimens used, predominantly oxaliplatin combined with fluoropyrimidine or other chemotherapeutic agents. Furthermore, CEA was measured in ng/L with normal values below 4.7 ng/L, and CA199 in U/mL with normal values below 27 U/mL at the time of initial diagnosis. This normal reference range for CA199 (≤27 U/L) is the standardized clinical testing criterion of the participating institutions, consistent with the chemiluminescent immunoassay platform used for digestive system malignancies during the study period.

### 2.3 Treatment and assessment

Patients in this study were categorized into two groups based on adjuvant chemotherapy status: those who underwent the treatment and those who did not. The primary adjuvant chemotherapy regimens administered were as follows: 1) modified FOLFOX6: fluorouracil 2400mg/m^2^ continuous infusion following a 400mg/m^2^ or 600mg/m^2^ IV bolus, leucovorin 400 mg/m^2^, and oxaliplatin 85 mg/m^2^ every two weeks. 2) capecitabine and oxaliplatin: capecitabine at 1,000 mg/m^2^ twice daily from day 1 to 14, repeated every three weeks, oxaliplatin 130 mg/m^2^ every three weeks. 3) S-1 and oxaliplatin: S-1S-1 at 80 mg/m^2^ from day 1 to 14, repeated every three weeks, oxaliplatin at 85 mg/m^2^ every two weeks. During adjuvant therapy, treatment efficacy was evaluated every four cycles. After the completion of therapy, assessments were conducted every 3 to 6 months for the first two years, and annually thereafter. Patients experiencing any discomfort could undergo additional evaluations at any time. These follow-up visits included a physical examination and routine imaging assessments, such as chest, abdominal, and pelvic CT scans to check for signs of health deterioration, recurrence or metastasis. RFS was defined as the time from surgery to recurrence (local/distant), death from any cause, or last imaging follow-up (whichever occurred first). OS was calculated from resection to death or last confirmed survival status. We applied standard right-censoring in Kaplan-Meier analyses. For RFS: Patients without recurrence/metastasis and alive were censored at the last imaging confirmation date. For OS: Patients alive at data cutoff were censored at the last follow-up contact (e.g., clinic visit or telephone confirmation).

### 2.4 Statistical analysis

Patients were stratified based on whether they received adjuvant chemotherapy, and baseline characteristics were presented using descriptive statistics, with categorical variables summarized by frequency counts. Missing data were non-randomly distributed due to incomplete retrospective clinical records. Regression imputation was used to handle missing values for key clinicopathological variables. Comparisons between categorical groups were conducted using the Chi−square test. Fisher’s exact test was used when the expected frequency was less than 1. Continuous variables were compared using the Wilcoxon rank-sum test where appropriate. Survival curves were visualized and estimated using the Kaplan-Meier method, with differences between the curves assessed using log-rank tests. Cox proportional hazards regression models were applied to assess the association between patient characteristics and time-to-event outcomes while controlling for covariates. A backward elimination technique was used to build multivariable models for certain outcomes in which factors that showed univariate associations at P < 0.1 were passed to a second stage for multivariable modeling. In the second stage, all variables were placed in the model and the least significant factors were removed until only factors with P <0.05 were left in the model. Stratified and subgroup analyses were carried out on the variables from the multivariate analysis to pinpoint the groups that benefited most from adjuvant chemotherapy. For all analyses, a P value of < 0.05 was considered statistically significant. All statistical evaluations were executed using SPSS 27.0 (SPSS Inc., Chicago, IL, USA).

## 3 Results

### 3.1 Patient characteristics

From January 1998 to December 2021, we analyzed 98 DA patients: 61 males and 37 females, aged 35-75 years (median age 55). Tumor location was distributed as D1 (n=6), D2 (n=70), D3 (n=8), and D4 (n=14). A total of 61 patients underwent pancreaticoduodenectomy (PD), while 28 patients had segmental resection (SR). Additionally, there were 9 cases where the surgical approach was not clearly documented. 45 received surgical resection and adjuvant chemotherapy, while 53 had surgery alone. Staging (AJCC 8th edition) showed 8 at stage I, 5 at IIA, 38 at IIB, 18 at IIIA, and 17 at IIIB. Most (75) were T3-T4, and 10 were T1-2. Nodal involvement was N0 in 46, N1 in 18, and N2 in 17. Of 88 patients with histological grading, 10 had well-differentiated, 50 moderately differentiated, and 28 poorly differentiated tumors. Guidelines recommend examining at least 8 regional lymph nodes; 50 patients met this, and 29 did not. Elevated CEA was found in 34 patients, and CA199 in 42. Adjuvant chemotherapy included fluoropyrimidines plus oxaliplatin in 33 patients, with 12 receiving other agents. Baseline characteristics were comparable between groups (P > 0.05) (**Table 1**). Consistent with our study’s enrollment criteria (which excluded patients with severe postoperative complications precluding timely adjuvant chemotherapy), no in-hospital or 30-day postoperative mortality was observed in the cohort, and no significant postoperative morbidity was documented among all included patients.

**Table 1:** Baseline characteristics of DA patients - adjuvant chemotherapy vs. no chemotherapy.

Characteristics	Stratification	All patients	Adjuvant chemotherapy	No chemotherapy	p-value
		n=98	n=45	n=53	
Age(year)	Median(range)		55(41-73)	54(34-75)	0.91
	<45	15	8	7	0.44
	45-65	63	29	34	
	>65	20	8	12	
Gender	male	61	31	30	0.21
	female	37	14	23	
ECOG	0	24	11	13	0.52
	1	33	18	15	
	NA	57	16	25	
TNM stage	1	8	2	6	0.11
	2A	5	2	3	
	2B	38	15	23	
	3A	18	13	5	
	3B	17	8	9	
	NA	12	5	7	
T stage	T1	3	1	2	0.66
	T2	7	4	3	
	T3	14	8	6	
	T4	61	27	34	
	NA	13	5	8	
N stage	N0	46	19	27	0.42
	N1	18	12	6	
	N2	17	8	9	
	NA	17	6	11	
Histology	WD	10	3	10	0.35
	MD	50	27	23	
	PD	28	13	15	
	NA	10	2	8	
Lymphatic/vascular invasion	YES	16	8	8	0.77
	NO	74	34	40	
	NA	8	3	5	
Perineural invasion	YES	11	5	6	0.93
	NO	79	37	42	
	NA	8	3	5	
Lymph nodes examined	<8	29	11	18	0.17
	≥8	50	27	23	
	NA	19	7	12	
CA199(U/mL)	>27	42	21	21	0.92
	≤27	43	22	21	
	NA	13	2	11	
CEA(ng/L)	>4.7	34	17	17	0.93
	≤4.7	53	27	26	
	NA	11	1	10	
Tomor location	D1	6	3	3	0.01
	D2	70	24	46	
	D3	8	8	0	
	D4	14	10	4	
Surgery procedure	whipple	61	21	40	0.01
	Segmental	28	19	9	
	NA	9	4	5	

### 3.2 Adjuvant chemotherapy for DA

The median follow-up was 49 months (range 15-80). Of the cohort, 45 patients (45.92%) received adjuvant chemotherapy. The most common regimens were modified FOLFOX6 (n=12), capecitabine plus oxaliplatin (n=11), and S-1 plus oxaliplatin (n=7). Other regimens included various combinations of fluorouracil, gemcitabine, oxaliplatin, and other agents (n=15). Kaplan-Meier analysis showed no significant differences in median OS (36.5 vs. 55.9 months, p=0.86) or RFS (38.2 vs. 39.4 months, p=0.60) between oxaliplatin and fluoropyrimidine -based regimens and other chemotherapy regimens.

### 3.3 The univariate and multivariate COX analysis for RFS

**Figure 1:**
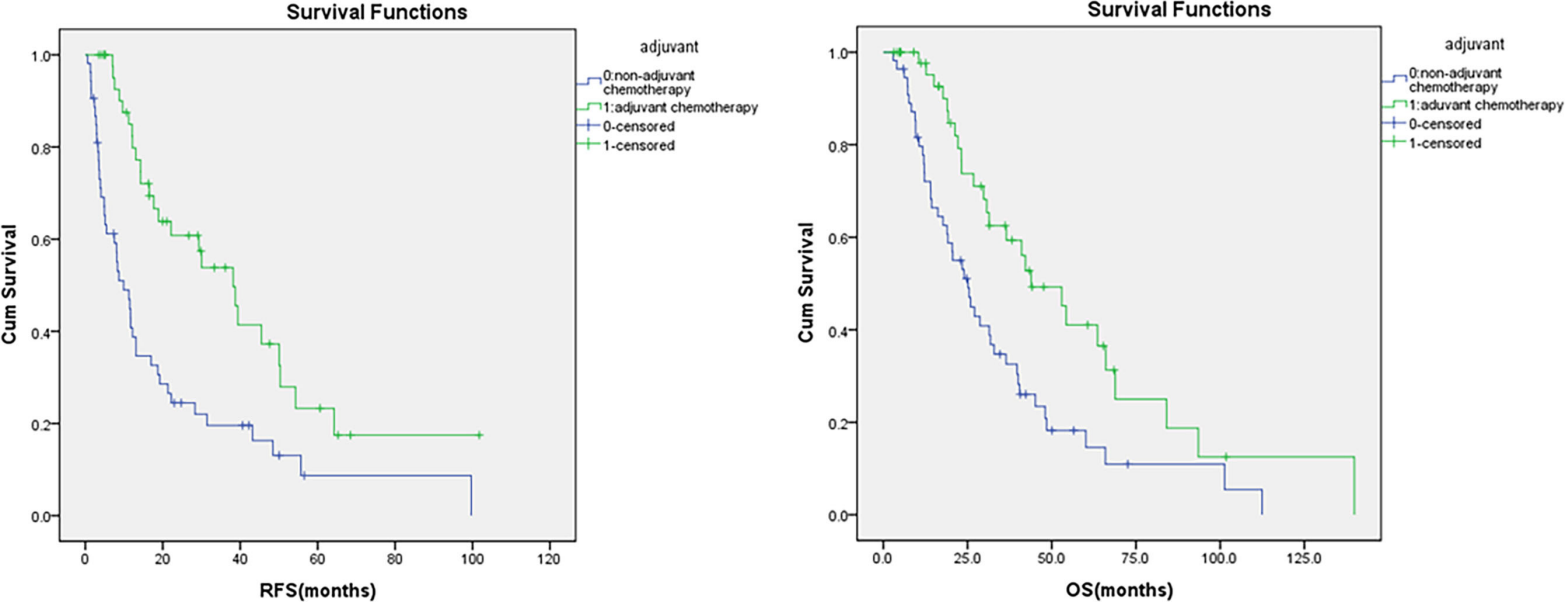
Kaplan-Meier curves for RFS and OS according to adjuvant chemotherapy status.

**Figure 2:**
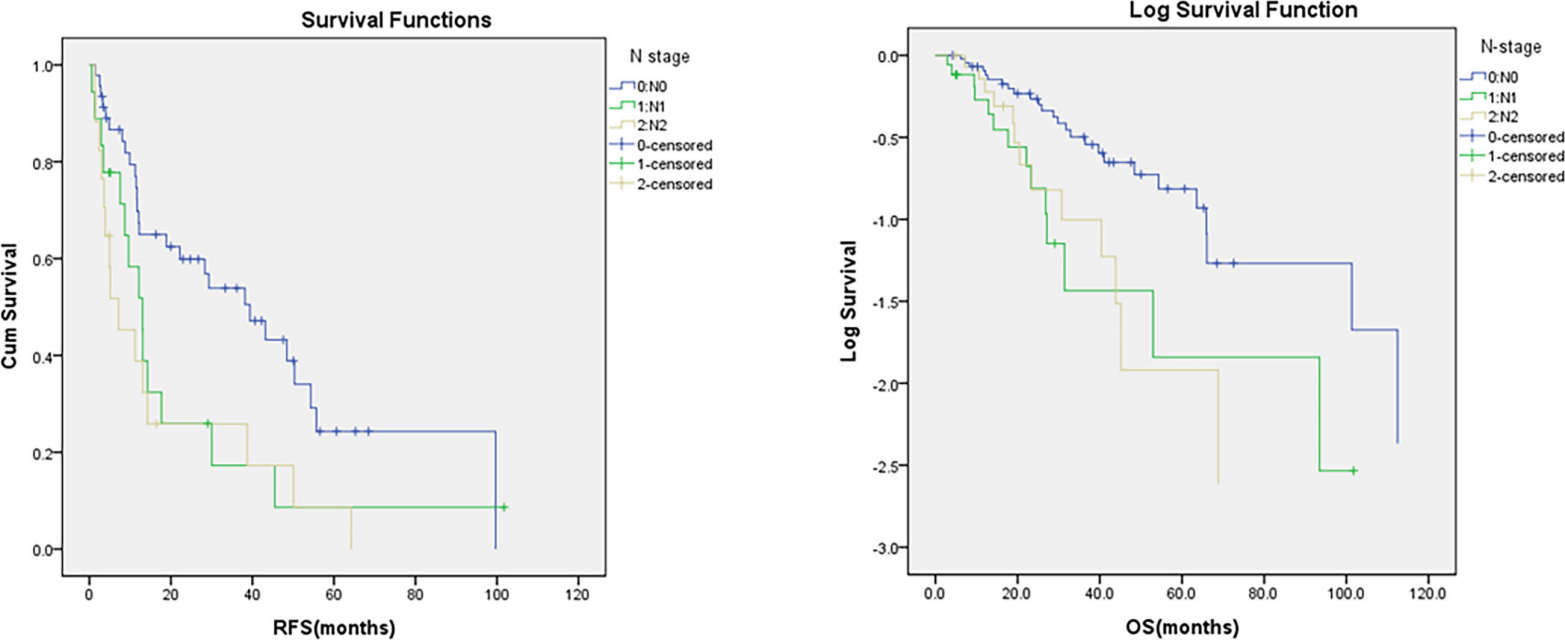
Comparison of RFS and OS by the status of N stage.

**Figure 3:**
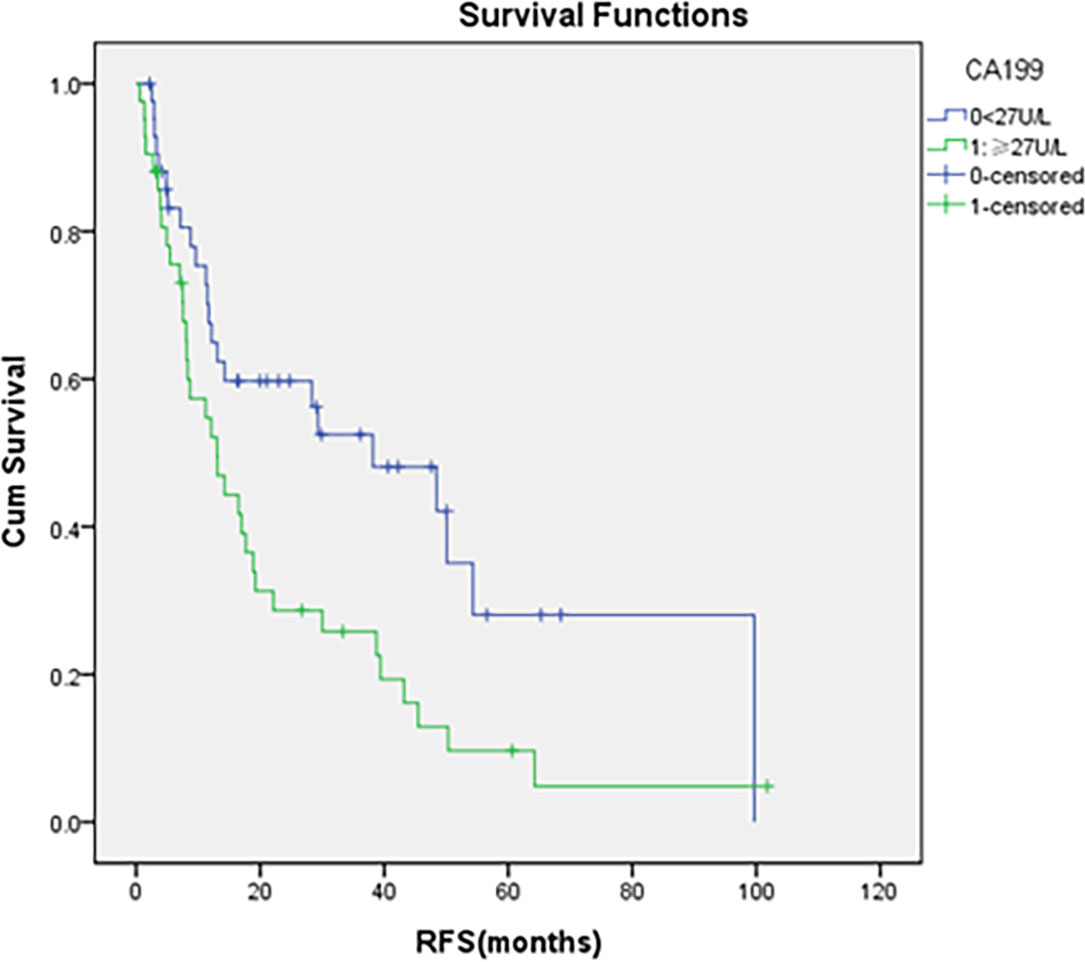
Comparison of RFS by the status of preoperative CA199 levels.

During a median follow-up of 49 months (range 15-80), 70.4% of patients (69 individuals) experienced recurrence or metastasis, with an average RFS of 13.0 months. Univariate Cox regression showed that patients receiving adjuvant chemotherapy had longer RFS than those who did not (38.2 vs. 9.9 months, p<0.001) (**Figure 1**). N stage (N0, N1, N2) was significantly associated with worse RFS (39.4 vs. 13.1vs. 7.1 months, p=0.01) (**Figure 2**), as were elevated preoperative CA19-9 levels (38.2 vs. 13.1 months, p=0.01) (**Figure 3**) and TNM stage (p=0.05). Other factors like age, gender, ECOG, histological grade, T stage, perineural invasion, and preoperative CEA were not significantly associated with RFS (p>0.5).

In the multivariate Cox model, adjuvant chemotherapy was linked to longer RFS (HR = 0.24, 95% CI: 0.12-0.46, p<0.001), while N stage (HR = 1.93, 95% CI: 1.33-2.79, p<0.001) and elevated CA19-9 levels (HR = 1.98, 95% CI: 1.06-3.71, p=0.03) were associated with worse RFS. Age had no significant impact on RFS (HR = 1.35, 95% CI: 0.82-2.23, p=0.24).

### 3.4 The univariate and multivariate COX analysis for OS

**Figure 4:**
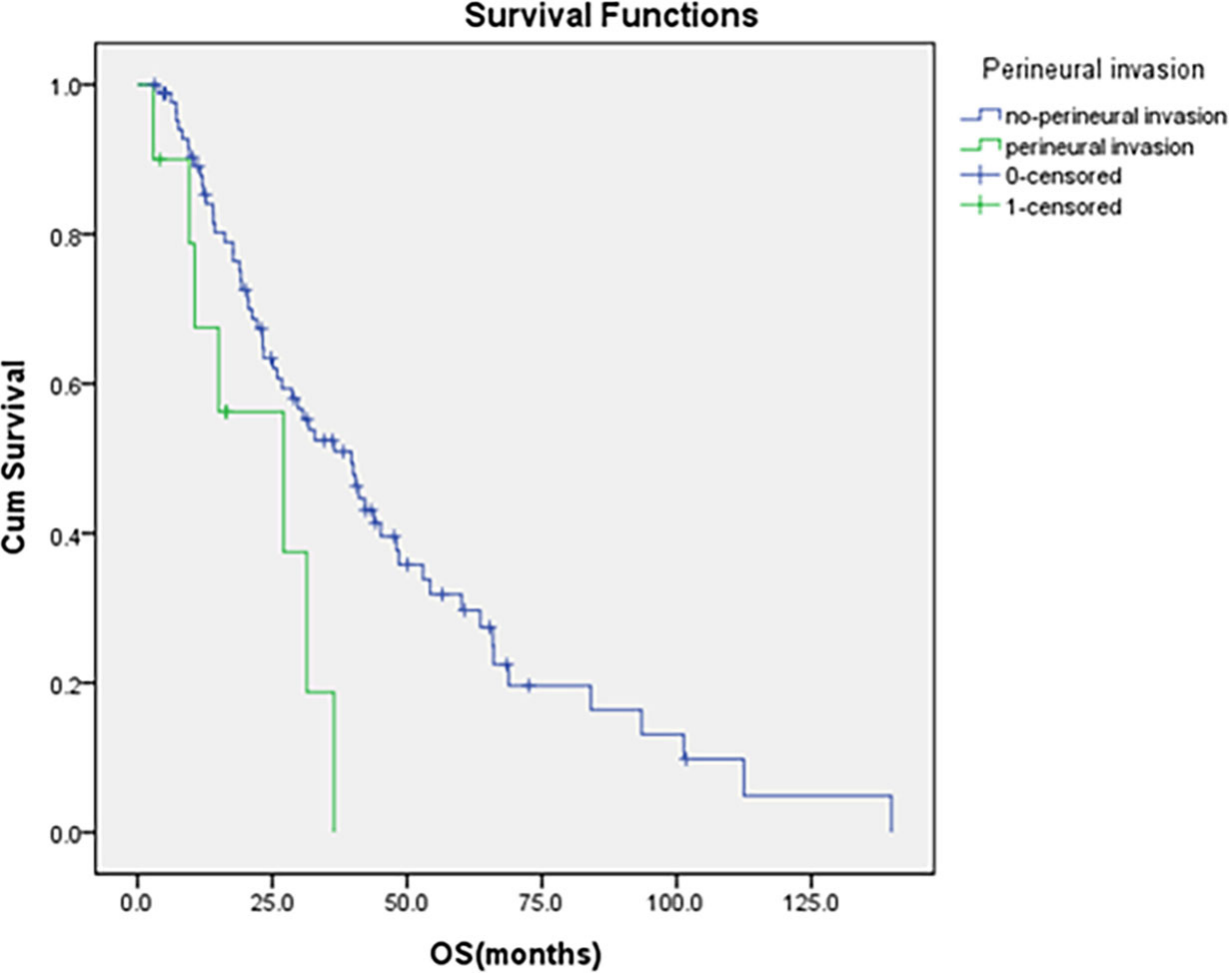
Comparison of OS by the status of perineural invasion.

The median OS was 24.9 months. Univariate Cox analyses showed that patients receiving adjuvant chemotherapy had significantly longer OS than those who did not (52.9 vs. 25.1 months, p=0.003) (**Figure 1**). N stage (N0, N1, N2) was significantly associated with poorer OS (48.4 vs. 23.2vs. 20.4months, p=0.01) (**Figure 2**), and perineural invasion also correlated with reduced OS (27.1 vs. 39.7 months, p=0.01) (**Figure 4**). Other factors, such as age, gender, ECOG, histological grade, TNM stage, T stage, lymphatic/vascular invasion, number of lymph nodes examined, tumor location (D1–D4), surgical approach (Whipple vs. isolated duodenal resection), and elevated preoperative CA19-9 and CEA levels, were not significantly associated with OS (p>0.5) (**Table 2**).

**Table 2:** Univariate Cox analysis - factors affecting PFS& OS in DA patients.

Characteristic	PFS			OS		
	mPFS	95%CI	p-value	mOS	95%CI	p-value
Age			0.06			0.13
<45	14.2	10.6-17.8		23.4	19.1-27.7	
45-65	22.2	6.3-38.1		40.0	27.5-52.4	
>65	11.2	3.6-18.8		27.1	13.7-40.5	
Gender			0.51			0.50
male	16.5	10.6-22.4		30.7	23.9-37.5	
remale	17.7	4.7-30.7		36.4	20.3-52.6	
ECOG			0.73			0.18
0	12.2	9.8-14.6		23.2	18.4-27.9	
1	12.1	5.1-19.1		31.4	28.6-34.2	
TNM stage			0.049			0.1
I	18.9	14.3-23.5		31.8	30.9-32.6	
IIA	29.3	14.1-44.5		39.7	18.5-60.9	
IIB	28.3	0.0-61.2		36.5	19.1-53.8	
IIIA	17.7	8.9-26.5		31.4	23.0-39.7	
IIIB	7.1	0.0-15.0		19.3	16.5-22.0	
Histology grade			0.31			0.68
Well differentiated	12.2	0.0-27.3		25.9	9.4-42.4	
Moderately differentiated	19.2	0.7-37.7		31.4	16.3-46.5	
Poorly differentiated	9.6	6.3-12.9		28.7	12.1-45.3	
T Stage			0.74			0.42
T1	17.0	–		60.1	–	
T2	12.2	1.4-23.0		29.8	20.8-38.8	
T3	17.7	3.1-32.3		45.2	24.9-65.4	
T4	12.1	10.5-13.7		27.1	16.7-37.5	
N Stage			0.01			0.01
N0	39.4	21.8-57.0		48.4	26.3-70.5	
N1	13.1	8.7-17.5		23.2	12.6-33.9	
N2	7.1	0.0-15.0		20.4	12.9-28.0	
Tomor location			0,2			0.5
D1	22.6	8.5-35.7		36.4	30.4-42.5	
D2	11.7	9.7-13.7		26.9	20.0-33.7	
D3	52.7	34.2-71.2		52.8	33.1-71.1	
D4	17,7	10.4-69.1		40.4	33.8-46.9	
Surgery procedure			0.9			0.5
Whipple	16.5	6.5-26.5		31.4	24,6-38.2	
Segmental resection	18.9	10,5-27.2		41.0	31.6-60.4	
NA	13.1	3.1-23,1		14.1	3.6-24.5	
Perineural invasion			0.13			0.01
YES	12.1	6.0-18.3		27.1	9.2-45.0	
NO	17.7	8.5-26.9		39.7	29.0-50.3	
Lymphatic/vascular invasion			0.58			0.49
YES	11.2	6.2-16.3		31.4	11.1-51.8	
NO	17.7	9.2-26.2		32.9	21.2-44.6	
lymph nodes examined			0.81			0.86
<8	17.7	3.7-31.7		30.7	24.3-37.2	
≥8	13.1	0.0-31.7		31.8	15.7-47.8	
CA199(U/mL)			0.01			0.05
<27	38.2	13.7-62.6		41.0	21.5-60.6	
≥27	13.1	6.4-19.8		26.8	16.7-37.0	
CEA (ng/ml)			0.55			0.87
<4.7	13.1	9.74-16.5		31.4	19.2-43.6	
≥4.7	28.3	12.4-44.2		29.8	5.5-54.1	
Adjuvant chemotherapy			0			0
YES	38.2	27.4-49.0		52.9	32.6-73.3	
NO	9.9	5.3-14.5		25.1	18.1-32.1	
Chemotherapy regiments			0.99			0.85
Fluoropyrimidines+Oxaliplantin	30.0	6.3-53.7		36.5	0.0-74.7	
Other	39.4	15.0-64.3		54.3	34.4-74.2	

In the multivariate model, adjuvant chemotherapy was linked to longer OS (HR = 0.47, 95% CI: 0.26-0.85, p=0.01), while N stage (HR = 1.70, 95% CI: 1.20-2.42, p=0.003) and perineural invasion (HR = 2.71, 95% CI: 1.14-6.44, p=0.03) were associated with poorer OS. Preoperative CA19-9 levels did not significantly affect OS (HR = 1.12, 95% CI: 0.60-2.10, p=0.72). In detail, adjuvant chemotherapy correlates with a 76.2% decrease in recurrence risk (HR = 0.24, 95%CI:0.12-0.46, p<0.001) and a 53.3% decrease in mortality risk (HR = 0.47, 95%CI:0.26-0.85, p=0.01).

### 3.5 Stratification and subgroup analysis impact of adjuvant chemotherapy

To potential trends subgroups that might benefit from adjuvant chemotherapy, we used multivariate Cox analysis for stratification based on TNM stage, N stage, CA199 levels, and perineural invasion. In stages IIIA and IIIB, exploratory analysis suggested patients receiving adjuvant chemotherapy had longer RFS (30.0 vs. 2.9 months, p=0.003; 14.2 vs. 3.6 months, p=0.001, respectively), representing a preliminary observation. Stage IIB patients also demonstrated a trend toward improved RFS with adjuvant chemotherapy (50.3 vs. 11.5 months, p=0.06), while no apparent RFS differences were found in stages I and IIA (**Table 3**).

**Table 3:** Comarrison of effetion on adjuvant chemotherapy analysis stratified.

Outcome	Stratify	Adjuvant chemotherapy	No chemotherapy	Chi-square	P value
		Median(mo) (95%CI)	Median(mo) (95%CI)		
RFS	stage I	18.9(0.0-40.1)	17.00(-)	0.00	0.99
	stage IIA	29.3(-)	22.2(7.6-36.8)	1.18	0.28
	stage IIB	50.3(25.3-75.3)	11.5(6.2-16.8)	3.66	0.06
	stage IIIA	30.0(6.1-53.9)	2.9(0.0-6.3)	8.90	0.00
	stage IIIB	14.2(8.8-19.6)	3.6(1.8-5.4)	11.43	0.00
OS	stage I	23.2(-)	31.8(0.0-66.2)	1.04	0.31
	stage IIA	29.8(-)	39.7(0.0-80.6)	0.56	0.45
	stage IIB	63.6(37.8-89.4)	25.9(13.2-38.6)	3.13	0.08
	stage IIIA	52.9(17.9-87.9)	9.6(0.0-21.6)	5.71	0.02
	stage IIIB	30.7(11.6-49.8)	18.9(4.4-32.5)	1.20	0.27
RFS	stage N0	50.3(21.4-79.2)	12.2(0.0-29.1)	3.88	0.05
	stage N1	14.2(7.1-21.4)	2.9(0.4-5.4)	11.29	0.00
	stage N2	38.7(0.0-89.0)	3.6(1.8-5.4)	13.23	0.00
OS	stage N0	63.6(51.1-76.1)	32.9(21.1-44.7)	4.10	0.04
	stage N1	26.8(15.6-38.1)	9.4(2.7-16.1)	8.99	0.00
	stage N2	43.9(16.4-71.3)	18.9(5.4-32.5)	3.41	0.07
RFS	CA199<27U/mL	50.1(23.8-76.3)	11.7(8.194-15.2)	2.53	0.11
	CA199≥27U/mL	22.1(0.0-45.2)	7.5(3.3-11.7)	18.20	0.00
OS	CA199<27U/mL	54.3(18.5-90.1)	28.7(18.6-38.8)	2.88	0.09
	CA199≥27U/mL	36.5(12.2-60.8)	18.9(9.6-28.3)	3.99	0.05
RFS	Perineural invasion(NO)	38.7(24.7-52.7)	11.2(7.0-15.5)	9.30	0.00
	Perineural invasion(YES)	30.0(-)	4.9(0.0-11.1)	6.73	0.01
OS	Perineural invasion(NO)	54.3(30.8-77.8)	25.9(12.9-38.9)	6.38	0.01
	Perineural invasion(YES)	31.4(-)	10.7(5.1-16.0)	0.99	0.32

For OS, exploratory analysis indicated stage IIIA patients receiving adjuvant chemotherapy had longer median OS (52.9 vs. 9.6 months, p=0.02), while stage IIB showed a trend towards improved OS (63.6 vs. 25.9 months, p=0.08). However, no significant OS differences were observed in stages I, IIA, and IIIB.

Subgroup analyses were conducted to further explore potential trends in the effects of adjuvant chemotherapy on OS and RFS among different subgroups, with a preliminary focus on investigating possible patterns of benefit from adjuvant chemotherapy. The comprehensive results are detailed in **Table 4**.

**Table 4:** Subgroup analysis - impact of adjuvant chemotherapy on RFS and OS.

Variable	Group	RFS			OS		
		HR	95%CI	P-value	HR	95%CI	P-value
Stage	I	1.02	0.11-9.86	0.99	3.87	0.24-63.34	0.34
	IIA	0.30	0.03-2.98	0.30	0.43	0.04-4.20	0.47
	IIB	0.42	0.17-1.05	0.06	0.45	0.18-1.12	0.08
	IIIA	0.18	0.05-0.63	0.01	0.25	0.07-0.84	0.03
	IIIB	0.13	0.03-0.49	0.00	0.54	0.18-1.64	0.28
N Stage	N0	0.44	0.19-1.02	0.06	0.42	0.18-1.00	0.05
	N1	0.14	0.04-0.52	0.00	0.19	0.06-0.63	0.01
	N2	0.09	0.02-0.42	0.00	0.34	0.10-1.13	0.08
CA199(U/mL)	<27	0.51	0.22-1.19	0.12	0.48	0.20-1.14	0.10
	≥27	0.22	0.11-0.47	0.00	0.51	0.26-1.00	0.05
PerineuralInvasion	NO	0.44	0.26-0.76	0.00	0.50	0.29-0.87	0.01
	YES	0.09	0.01-0.81	0.03	0.44	0.89-2.30	0.33

The analysis revealed that adjuvant chemotherapy appeared to improve median RFS in DA patients at TNM stages IIIA and IIIB (HR = 0.18, 95%CI=0.05-0.63, p=0.01; HR = 0.13, 95%CI = 0.03-0.49, p = 0.003, respectively). Notably, only stage IIIA patients demonstrated a suggestive improvement in median OS (HR = 0.25, 95% CI = 0.07-0.84, p = 0.03). For N-stage N1 and N2 patients, adjuvant chemotherapy was associated with apparent improvements in median RFS (HR = 0.14, 95%CI = 0.04-0.52, p = 0.003; HR = 0.09, 95%CI = 0.02-0.42, p = 0.00, respectively), while only N-stage N1 showed a showed a trend toward improved median OS (HR = 0.19, 95%CI = 0.06-0.63 p = 0.01). while N2 stage exhibited no clear association with improved median OS (HR = 0.34, 95%CI = 0.10-1.13, p = 0.08). Both subgroup and stratified analyses indicated that DA patients with initial CA199 levels≥ 27U/mL who received adjuvant chemotherapy had seemingly prolonged median RFS (22.1 vs. 7.5 months, p<0.001) and OS (36.5 vs. 18.9 months, p=0.05), alongside apparent reductions in risks of recurrence (HR = 0.22, 95%CI: 0.11-0.47, p<0.001), and death (HR = 0.51, 95%CI: 0.26-1.00, p=0.05) than those who did not receive adjuvant chemotherapy. DA patients with or without perineural invasion who received adjuvant chemotherapy also displayed suggestive improvements in RFS and OS (**Table 3**).

## 4 Discussion

Localized DA is primarily managed through surgical intervention. However, a significant number of patients subsequently experience local and distant recurrences, often leading to mortality. Research indicates that approximately 56% of patients who undergo surgery for SBA suffer from relapse or metastasis (5). This high recurrence rate highlights the potential role of adjuvant chemotherapy in addressing micrometastases and minimizing recurrence. Given the rarity of DA, there is a notable scarcity of prospective clinical guidelines. The benefits of adjuvant chemotherapy for DA mainly arise from retrospective studies, and the 2020 NCCN Small Bowel Adenocarcinoma Guidelines (Version 1.2020) formally endorse adjuvant chemotherapy for stage III patients and advise its consideration for high-risk stage II as a Category IIA recommendation—this guideline standardized clinical practice for the later period of our 1998–2021 study cohort. Retrospective evaluations and National Cancer Database (NCDB) analyses on the efficacy of SBA adjuvant chemotherapy (including DA) offer varied results. Some evidence supports the benefits of adjuvant chemotherapy (6, 7), while others contradict (8–11). However, the absence of top-tier evidence—particularly randomized controlled trials (RCTs)—has perpetuated ongoing controversy regarding the role of adjuvant chemotherapy in post-resection DA management. Our retrospective study demonstrates that adjuvant chemotherapy independently improves both RFS and OS, thereby providing critical retrospective evidence to inform this unresolved clinical debate.

Notably, our reported median OS of 24.9 months is indeed lower than the approximately 30 months reported in the literature, which also indicates a 5-year survival rate of 60-70% (4). Studies have identified poor prognostic indicators such as male gender, advanced overall tumor stage, late T stage, lymph node involvement, poor tumor differentiation, and elevated preoperative CA199 and CEA levels (4, 12). In our study, 62.2% of the patients were male, 74.5% were stage IIB or higher, 62.2% had T4 tumors, and 79.6% of tumors were moderately to poorly differentiated. Additionally, some patients remained alive at the end of the follow-up period, which may have influenced the overall results.

The staging of Small Bowel Adenocarcinoma (SBA) is analogous to that of Colorectal Cancer (CRC), with the prognosis being closely linked to the stage of the disease (12). Numerous studies have established a correlation between the TNM staging of advanced small bowel cancer and the 5-year OS and RFS rates (5, 13). Earlier research has identified the TNM stage as a critical determinant of OS (14, 15). In our study, the TNM stage adversely affected RFS, consistent with prior reports. We utilized TNM staging to identify those who might benefit more from adjuvant chemotherapy in DA patients. Stratified and subgroup analyses showed that stage IIIA and IIIB patients benefited from adjuvant chemotherapy in terms of RFS, with stage IIB patients showing a promising trend. Multivariable COX analysis suggested an improved OS with adjuvant chemotherapy for certain patient groups. Notably, for stage IIIA DA patients, both subgroup and stratified analyses showed that adjuvant chemotherapy increased OS and decreased mortality. Kristian K et al.’s findings align with ours, demonstrating reduced mortality with adjuvant chemotherapy (HR 0.29, 95%CI: 0.11–0.76, P = 0.01) (16). Prior studies have indicated that adjuvant chemotherapy improves survival in patients with resected stage III SBA (6, 17). Although we observed an extension in OS among stage IIIB DA patients receiving adjuvant chemotherapy, this difference did not reach statistical significance, possibly due to our limited sample size and a substantial number of patients not reaching the study endpoint. As a retrospective study with patient enrollment spanning 1998–2021, the administration of adjuvant chemotherapy was determined by clinical decision-making rather than randomization, an inherent limitation compounded by the evolving clinical guidance for DA over our long study period. The NCCN Small Bowel Adenocarcinoma Guidelines (Version 1.2020) only formally endorsed adjuvant chemotherapy for stage III DA as a Category IIA recommendation in 2020—prior to this, clinical practice relied on extrapolation from colorectal cancer, with decision-making further complicated by the unique clinical challenges of DA: patients undergoing pancreaticoduodenectomy have significant postoperative physical burden, and eligibility for adjuvant chemotherapy required assessment of postoperative general condition, performance status, patient willingness, and complication status. These factors collectively resulted in 14 stage III DA patients in our cohort not receiving adjuvant chemotherapy, and this real-world clinical variability may affect the interpretability of stage III subgroup analyses.

DA patients with stage III seem to experience more substantial benefits from adjuvant chemotherapy, with those at stage IIB showed a tendency toward a reduced recurrence likelihood. Given these observations, it is advisable to advocate for adjuvant chemotherapy in stage III DA patients. Furthermore, dedicated research efforts should be intensified to explore the factors influencing the efficacy of chemotherapy in stage II DA patients, particularly to understand and enhance its potential benefits.

Lymph node involvement is a strong prognostic indicator supported by substantial evidence (15, 18). The N-stage, categorized into N0, N1, and N2, reflects the extent of regional lymph node involvement: N0 denotes no metastasis, N1 indicates the presence of 1–2 positive lymph nodes, and N2 represents 3 or more positive nodes. In our research, the N stage, including N0, N1, and N2, displayed a significant correlation with reduced RFS and OS. Further subgroup analysis suggested that patients with advanced N stages (N1, N2) tended to have poorer RFS and OS in DA patients. Corroborating our results, both meta-analyses and recent large-scale studies have confirmed the negative prognostic impact of lymph node involvement on small bowel cancer surviva (10, 19, 20). Thus, lymph node stages N1 and N2 appear indicative of less favorable outcomes in this exploratory analysis, suggesting that adjuvant chemotherapy may be a consideration worth evaluating for patients with positive regional lymph nodes.

Our findings also suggest that while adjuvant chemotherapy appeared to prolong OS in N1 patients((HR = 0.19, 95%CI = 0.06-0.63 p = 0.01), was not observed for N2 patients (HR = 0.34, 95%CI = 0.10-1.13, p = 0.08). This raises the possibility that, given the more advanced nodal involvement in N2 patients, relying solely on adjuvant chemotherapy may not be sufficient to counteract the aggressive nature of their disease in this patient cohort. N2 patients inherently have a poorer prognosis, and their high recurrence rates may limit the effectiveness of adjuvant therapy alone, making it difficult to achieve statistically significant improvements in survival. In this context, neoadjuvant chemotherapy may represent a promising strategy. By potentially downstaging tumors prior to surgery, neoadjuvant therapy might improve surgical outcomes and enhance the efficacy of subsequent adjuvant treatments. Drawing parallels with gastric cancer, where neoadjuvant treatments have shown greater benefits, it stands to reason that similar approaches might yield improved results for N2 duodenal cancer patients as well. However, it is crucial to highlight that there is currently a lack of robust evidence specifically addressing the role of neoadjuvant chemotherapy in duodenal cancer, as most treatment strategies are adapted from guidelines for gastric or colorectal cancers. Given the gaps in the literature, further research is essential to define the potential benefits and optimize treatment strategies for N2 patients.

Multiple factors are associated with a less favorable prognosis, including advanced age, a compromised performance status, decreased serum albumin levels, elevated levels of CEA or CA199, poorly differentiated tumor histology, and positive surgical margins (21).

In our study, elevated preoperative CA199 levels and the presence of perineural invasion were identified as independent predictors of adverse outcomes in DA. Specifically, an increase in preoperative CA199 was linked to a shorter RFS, while perineural invasion was associated with a reduced OS. These findings are consistent with prior studies. For example, Kenji Nakagawa identified CA199 as a standalone factor influencing RFS (22). In a similar vein, Kohei Nishio et al. pinpointed high preoperative CA199 levels as an adverse prognostic marker for DA (16). Our stratified and subgroup analyses suggested that DA patients with an initial CA199 levels of 27U/mL or higher who received adjuvant chemotherapy exhibited apparent improvements in RFS and OS in this exploratory assessment. The rationale behind this association is that elevated CA19-9 levels may indicate a more aggressive tumor biology or a higher tumor burden, which could necessitate additional treatment beyond surgery alone. While our data suggest a potential benefit of adjuvant chemotherapy for patients with elevated CA19-9, we acknowledge the need for larger prospective studies to further validate this preliminary observation in clinical practice. Thus, while adjuvant chemotherapy may not be universally necessary for all patients with elevated CA19-9, it may appear particularly relevant for those presenting with much higher levels. Consistent with numerous studies that have underscored the adverse impact of perineural and vascular invasion (23, 24), our research also identified perineural invasion as a detrimental factor. We identified perineural invasion as an independent predictor of OS in DA, significantly increasing the risk of mortality in DA. In our Kaplan-Meier analysis, which used perineural invasion as a stratification factor, it was observed that patients without perineural invasion who underwent adjuvant chemotherapy exhibited notably extended OS and RFS. This finding presents a deviation from the current National Comprehensive Cancer Network (NCCN) guidelines, which may be attributed to the limited cohort size of patients with perineural invasion in our study. This discrepancy underscores the need for more extensive studies with larger datasets to validate these findings and refine treatment guidelines.

Numerous chemotherapeutic protocols are in use for metastatic SBA, including DA. Fluorouracil-based therapies, either as monotherapy or in combination with a platinum derivative, are most commonly utilized. Studies have shown that combining fluorouracil-based treatments with a platinum analog significantly enhances survival compared to other chemotherapy options (25–27). Before the establishment of European and NCCN guidelines, DA chemotherapy was extrapolated from treatments for gastric cancer, colorectal cancer, or periampullary carcinoma. In our study, a combination of oxaliplatin and fluorouracil-based agents (e.g., capecitabine, fluorouracil, or S-1) was predominant, representing 73.33% of cases. However, our analysis did not reveal a distinct survival advantage of the oxaliplatin-fluorouracil combination over other therapeutic regimens for DA. This observation may be attributed to the limited size of our study cohort and potential variations among the groups compared.

The retrospective nature of this study, compounded by DA’s rarity and the prolonged case accrual period (1998-2021), resulted in inherent documentation inconsistencies. Key variables including tumor subsites (proximal/distal duodenum), surgical technique details, lymph node harvest counts, and pathological staging parameters (T stage missing in 13.3% [13/98], N stage in 17.3% [17/98]) demonstrated incomplete recording, which may introduce potential bias despite the use of regression imputation. The decision to administer adjuvant chemotherapy was based on clinical discretion rather than random assignment, and baseline differences between patients who received and did not receive adjuvant chemotherapy may exist despite adjustment with conventional multivariable Cox regression modeling. Propensity score matching (PSM) and inverse probability of treatment weighting (IPTW) are well-recognized rigorous methods to mitigate such selection bias in retrospective analyses; however, these sensitivity analyses could not be performed in our study due to the overall small sample size of the cohort and the presence of missing data for several key clinical variables. Both factors preclude the generation of well-matched covariate groups (for PSM) and the calculation of stable weight estimates (for IPTW), which are essential for the validity of these methods. A potential methodological limitation is the risk of immortal time bias, as the treatment group was defined as patients who completed at least 4 cycles of adjuvant chemotherapy. While this bias is theoretically notable, its practical impact is mitigated by the standard 4–6 week postoperative recovery window for adjuvant chemotherapy initiation in DA clinical practice, a fixed interval following pancreaticoduodenectomy. However, we were unable to model time to treatment initiation as a time-dependent covariate due to incomplete recording of exact chemotherapy initiation dates in our retrospective cohort. Thus, our results should be interpreted with caution. Additionally, several important clinicopathological factors were not available in the retrospective dataset, including tumor location (proximal vs. distal), MMR/MSI status, gastric/intestinal histological subtyping of DA, PD-L1 expression scores, HER2 status, and detailed postoperative complications. MMR/MSI testing, histological subtyping, and other key immunohistochemical detections were not routinely performed during the early study period, resulting in extensive missing data that precluded their inclusion; retrospective supplementary testing is also unfeasible due to unavailable or poor-quality archived tissue samples from early cases. The small sample size, particularly in subgroup analyses, limits the robustness and generalizability of these findings. The absence of standardized postoperative adjuvant treatment protocols during the study period resulted in inconsistencies in treatment regimens and cycles, further complicating the analysis. The potential bias arising from the decision to administer adjuvant chemotherapy, coupled with the absence of comprehensive data on confounding factors, may lead to skewed comparisons. The insights provided are limited by the fact that our research is based on the experiences of two institutions, utiimpacting the reliability of adjusted survival rates across different disease stages and chemotherapy regimens. This variability in treatment methodologies and the lack of detailed data also precluded a thorough bias analysis in our study.

## 5 Summary

In summary, our study serves as a pioneering investigation into the efficacy of adjuvant chemotherapy in treating DA, using rigorous stratification and subgroup analysis for exploratory assessment. The results suggest that such chemotherapy may extend RFS in patients with stage IIIA and IIIB DA, and may enhance OS in those with stage IIIA DA, as preliminary observations from our small cohort. Notably, patients with DA exhibiting positive N stage, especially those with adjuvant pathological lymph node metastasis, appear to show potential benefit from adjuvant chemotherapy. Furthermore, our findings indicate a tentative trend that patients with elevated preoperative CA199 levels may also experience improved outcomes with this treatment approach in our study population. This research is vital, providing an in-depth analysis of the effects of adjuvant chemotherapy for DA, a topic previously characterized by limited data and a lack of clear consensus. By delivering robust and conclusive evidence on the role of adjuvant chemotherapy in DA, our study fills a critical gap in existing literature and holds the potential to significantly influence future clinical practices and research trajectories. Future investigations should focus on identifying specific chemotherapy regimens and implementing detailed stratification based on prognostic factors, thereby enhancing the precision and efficacy of DA treatments.

## Data Availability

The raw data supporting the conclusions of this article will be made available by the authors, without undue reservation.
